# Effects of rumen bypass melatonin feeding (RBMF) on milk quality and mastitis of Holstein cows

**DOI:** 10.7717/peerj.9147

**Published:** 2020-05-14

**Authors:** Songyang Yao, Hao Wu, Hui Ma, Yao Fu, Wenjuan Wei, Tiankun Wang, Shengyu Guan, Hai Yang, Xiubo Li, Jiangpeng Guo, Yongqiang Lu, Lu Zhang, Changwang He, Yi Chang, Guoshi Liu

**Affiliations:** 1 Beijing Key Laboratory of Animal Genetic Improvement, Key Laboratory of Animal Genetics and Breeding of the Ministry of Agriculture, National Engineering Laboratory for Animal Breeding, College of Animal Science and Technology, China Agricultural University, Beijing, China; 2 Beijing Shounong Animal Husbandry Development Co. LTD, Beijing, China; 3 Beijing Changping District Animal Disease Prevention and Control Center, Beijing, China; 4 Chinese Academy of Agricultural Sciences Feed Research Institute, Beijing, China; 5 Beijing Animal Husbandry Station, Beijing, China

**Keywords:** Melatonin, Mastitis, Inflammation, Milk quality

## Abstract

Cow mastitis is a major problem frequently encountered by dairy farmers and it is manifested by the high number of somatic cells and the low quality of the milk. The conventional treatment for mastitis is use of antibiotics. In the current study, a new approach is applied to target this disorder: rumen bypass melatonin feeding (RBMF). The RBMF significantly reduced milk somatic cell count and improved milk nutritional values with the elevated protein, fat and dry matter levels. This approach also suppresses the stress and proinflammatory responses of the cows indicated by the reduced serum cortisol, TNF-α and IL-6 and increased IL-10 levels. Importantly, the beneficial effects of RBMF have lasted for several days after termination of the treatment. The effects of melatonin on the mastitis are probably attributed to the antioxidant and anti-inflammatory activities of melatonin. Considering the none or low toxicity of melatonin to organisms and the no invasive nature of this approach, we recommend that RBMF could be used in large scale in the dairy farming to target the cow mastitis.

## Introduction

Dairy Herd Improvement (DHI) is an important index to evaluate the milk productive performance of a cow (Shock et al., 2015). DHI measurement includes somatic cell count (SCC), protein, fat, lactose, dry matter in the milk. The most important parameter to evaluate the quality of the milk is the SCC, which refers to the total number of somatic cells, immune-related lymphocytes and shed aging mammary epithelial cells per milliliter of milk. Actually, the white blood cells (WBC) account for 99% of the total number of somatic cells (Shook & Schutz, 1994). The numbers of SCC reflect the status of the udder health for the dairy cows (Caraviello et al., 2005). An increase in SCC is a substantial parameter that indicates potential mastitis in cows, which not only affects the production, but also reduces the nutrient value of the milk (Sert et al., 2016; Kul et al., 2019). At present, antibiotics are the conventional remedies for dairy cow mastitis(Fejzic et al., 2014). However, the long-term use of antibiotics leads to the rise of drug resistance in animals and, in addition, drug residues in the milk cause serious harm to human health as well as environmental pollution (Martinez, 2009). Thus, to identify the effective alternatives which can replace antibiotics is a good strategy to target the cow mastitis.

Melatonin (N-acetyl-5-methoxytryptamine) is an indolamine hormone (Reiter, 1991) which was first isolated from the pineal gland of cow by Lerner et al. (1958). Melatonin secretion exhibits a circadian rhythm with low level during the day and high level at night. Its peak level usually occurs at the middle of the night and the peak level is several times higher than that during the day in vertebrates (Reiter, 1993; Zhdanova et al., 1995). Melatonin is a pleiotropic molecule with many physio-pathological functions including its anti-stressful and anti-inflammatory activities, sleep promotion, mood improvement, reproductive regulation and immune enhancement functions (Brzezinski, 1997; Nabavi et al., 2019). In addition, melatonin is a potent free radical scavenger and antioxidant which is much more potent than that of classic antioxidants, vitamin E and vitamin C (Pieri et al., 1994). Melatonin can detoxify a variety of reactive oxygen species (ROS) and reactive nitrogen species (RNS) such as the highly toxic hydroxyl radical (OH), hydrogen peroxide (H_2_O_2_), superoxide anion (O_2_^−^), peroxynitrite anion (ONOO^−^), hypochlorous acid (HOCI), etc. to slow down the inflammatory response-induced tissue damages (Poeggeler et al., 1993; Pieri et al., 1994; Reiter, 2000; Reiter et al., 2001). It has been reported that subcutaneously melatonin injection not only reduces the milk SCC but also reduces serum cortisol, WBC, lymphocytes, serum IgG, IgM and increase albumin in the cows (Yang et al., 2017). The continuous injection has a potential risk of infection and it is also a stressful maneuver to the dairy cows. Obviously, the oral application of melatonin is the better method for melatonin delivery and this method has been successfully used in many animals (Kennaway & Seamark, 1980; Kennaway, Gilmore & Seamark, 1982; Bubenik & Smith, 1987; Zetner, Andersen & Rosenberg, 2016). However, cows are the ruminants and oral application melatonin needs to pass the rumen which may significantly decrease the melatonin’s bioavailability since the digestive enzymes and microbes in the rumen can metabolize melatonin. To avoid this obstacle, a new approach referred to as rumen bypass melatonin feeding (RBMF) was applied in the current study. The results are reported below.

## Materials & Methods

### Chemicals and agents

Melatonin was purchased from Sigma Aldrich (Burlington, Massachusetts, USA). The preparation of rumen bypass melatonin is made by our laboratory and Beijing Oriental Tianhe biotechnology co. LTD. Simply, rumen bypass melatonin preparation is composed of two parts including coating and nucleation. The coating is rumen-passing fat powder. Nucleation is a particle composed of the active ingredient melatonin and excipients. The excipients consisted of primary excipients of calcium stearate or silicon dioxide and the second excipients of starch, dextrin and sodium carboxymethyl cellulose. The rumen bypass melatonin was made up of melatonin (4%∼6%), the first admixture (4.4%∼4.6%), starch (21.52%∼22.34%), dextrin (15%∼17%), sodium carboxyformic acid cellulose (0.08%∼0.1%) and rumen fatty powder (49.96%∼55%). The rumen bypass ratio of RBMF is 83%∼85%.

### Animals

Experimental female cows were raised in semi-enclosed cowsheds with 23∼25 °C (both spray and fan were turned on to control the temperature) during the experiment. The cows were exposed to the natural light/dark cycles without human interfering and were free to feed. The variety of fodder was the lactating cow TMR daily ration (Table 1). All the experimental cows were adapted to the same program except for the artificial feeding of rumen bypass melatonin. The experimental animals were reviewed by the China Agricultural University Laboratory Animal Welfare and Animal Experimental Ethical Inspection Committee. The review number is AW20180502-1.

**Table 1:** TMR daily ration formula.

Feed name	Amount (kg/cow/day)
Megalac	0.130
PalmFatT	0.200
Beet Pulp Pellet	0.300
soybean hull	0.300
extruded soybean	0.400
Corn vapor pressure tablet310	4.100
Shounong high yield20191015	9.600
cotton seed with fluffy	2.000
Domestic oats	0.500
Imports of alfalfa	3.200
corn silage	19.500

### Study procedure

Holstein cows (*n* = 35) with high milk SCC (0.4∼0.7 million cells/ml) were randomly divided into three groups, two melatonin-treated groups with different doses of melatonin and a control group. One melatonin-treated group was feeding melatonin 40 mg/cow and another was 80mg/cow. Then the melatonin-treated cows were sub-grouped into melatonin-feeding 7-day group (40 mg group: *N* = 5, initial SCC average value: 0.432 million cells/ml. 80mg group: *N* = 5, initial SCC average value: 0.393 million cells/ml), 14-day group (40 mg group: *N* = 5, initial SCC average value: 0.412 million cells/ml. 80 mg group: *N* = 5, initial SCC average value: 0.406 million cells/ml) and 21-day group (40 mg group: *N* = 5, initial SCC average value: 0.489 million cells/ml. 80 mg group: *N* = 5, initial SCC average value:0.5 million cells/ml), respectively. The control group was also included 5 cows with no treatment (*N* = 5, initial SCC average value: 0.425 million cells/ml). The RBMF was carried out daily at 8:00 am. Simply, the prepared rumen bypass melatonin granules were quantitatively loaded into a paper package (digestible). For the deliverer, one hand opened the mouth of the cow from the side, and the other hand with a long arm glove deliver the rumen bypass melatonin paper package into the digestive duct around the part of the throat of the cow.

During the experiment, milk and blood were collected every two days at 9:00 am. Additionally, samples collection was also performed one week after RBMF termination in each group, respectively, to observe the lasting effect of this method of RBMF.

### Assay methods

DHI evaluation: the SCC was determined by Fossomatic™ FC (Serial No.91755377, Part No.60002326, made in Denmark) which was based on flow cytometry. Fossomatic FC counts somatic cells by the following steps. Milk was incubated in a unique culture medium, and then mechanically break up everything except the cells, whose colonies were also divided into an individual cell. During the culture, the cells are stained with a specific DNA-staining medium. At the measuring point, the red light was emitted by the stained cells when exposed to a beam of laser light source produced a pulse of light. The entire sample passed through the flow pool using a very precise syringe, and thus, cell passes one by one without stacking. The electronic device counts the pulses and displays them through the pulse height analysis diagram on the PC monitor and the numbers of cells were calculated and recorded.

Milk protein, fat, dry matter and lactose were measured by MilkoScan FT+(Serial No.91755049, Part No.60027086, made in Denmark) which was based on Fourier transform infrared spectrum analysis. MilkoScan™ FT+ operates in the mid-infrared region, with a spectral range of 3–10 µm corresponding to 1,000–5,000 cm^−1^. Fourier transform infrared spectrometer scans the whole infrared spectrum, collecting data and measuring new parameters. The assays were performed by the cow production performance laboratory of Beijing Animal Husbandry Station.

Melatonin and cortisol assays: melatonin assay kit was used to determine melatonin level by double antibody sandwich method followed by the manufacture’s instructions. Simply, the purified melatonin antibody was used to coat the microporous plate to make solid phase antibody. Melatonin was added to the microporous layer of the coated monoclonal antibody in turn, and then combined with HRP-labeled melatonin antibody to form the antibody-antigen-enzyme labeled antibody complex. After thorough washing, the substrate TMB was added for color development. TMB is converted to blue by HRP enzyme and to yellow by acid finally. The color depth was positively correlated with the melatonin in the sample. The absorbance (OD value) was measured with an enzyme marker at the wavelength of 450 nm, and the concentration of melatonin in the sample was calculated by the standard curve. The method of cortisol detection was similar to melatonin detection except for using cortisol antibody encased microporous plates.

Tumor necrosis factor alpha (TNF-α), interleukin-6 (IL-6) and interleukin-10 (IL-10) assays.

The methods of detecting TNF-α, IL-6 and IL-10 levels were similar to detecting melatonin except for using TNF-α antibody encased microporous plates, IL-6 antibody encased microporous plates and IL-10 antibody encased microporous plates, respectively.

The ELISA kits were purchased from Jiangsu Meimian Industrial Co., Ltd. (Jiangsu, China).

#### Statistical analyses

The data were expressed as mean ± SEM. The one-way ANOVA was used to evaluate the normality and followed by Duncan’s multiple tests for multiple comparisons. IBM SPSS Statistic v20 was used to conduct these analyses. *P* < 0.05 was considered as significant difference.

## Results

### Effects of RBMF feeding on milk DHI in Holstein dairy cows

#### Somatic cell count

**Figure 1:**
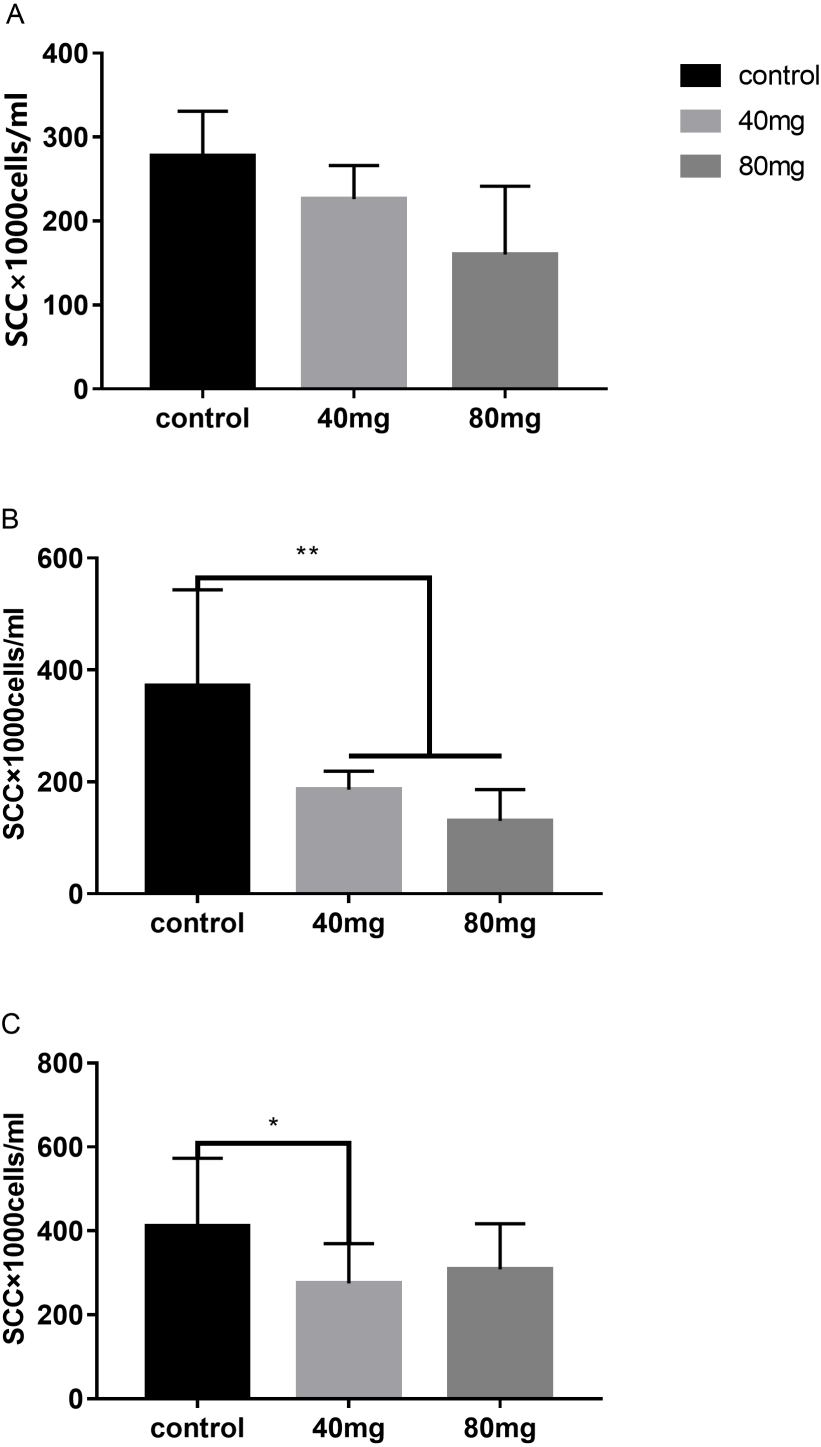
Effects of RBMF on the milk SCC in different groups. (A) The SCC in the RBMF 7-day group; (B) the SCC in the RBMF 14-day group; (C) the SCC in the RBMF 21-day group, respectively. Mean ±  SEM (*n* = 5), ^∗^*P* < 0.05, ^∗∗^*P* < 0.01.

**Figure 2:**
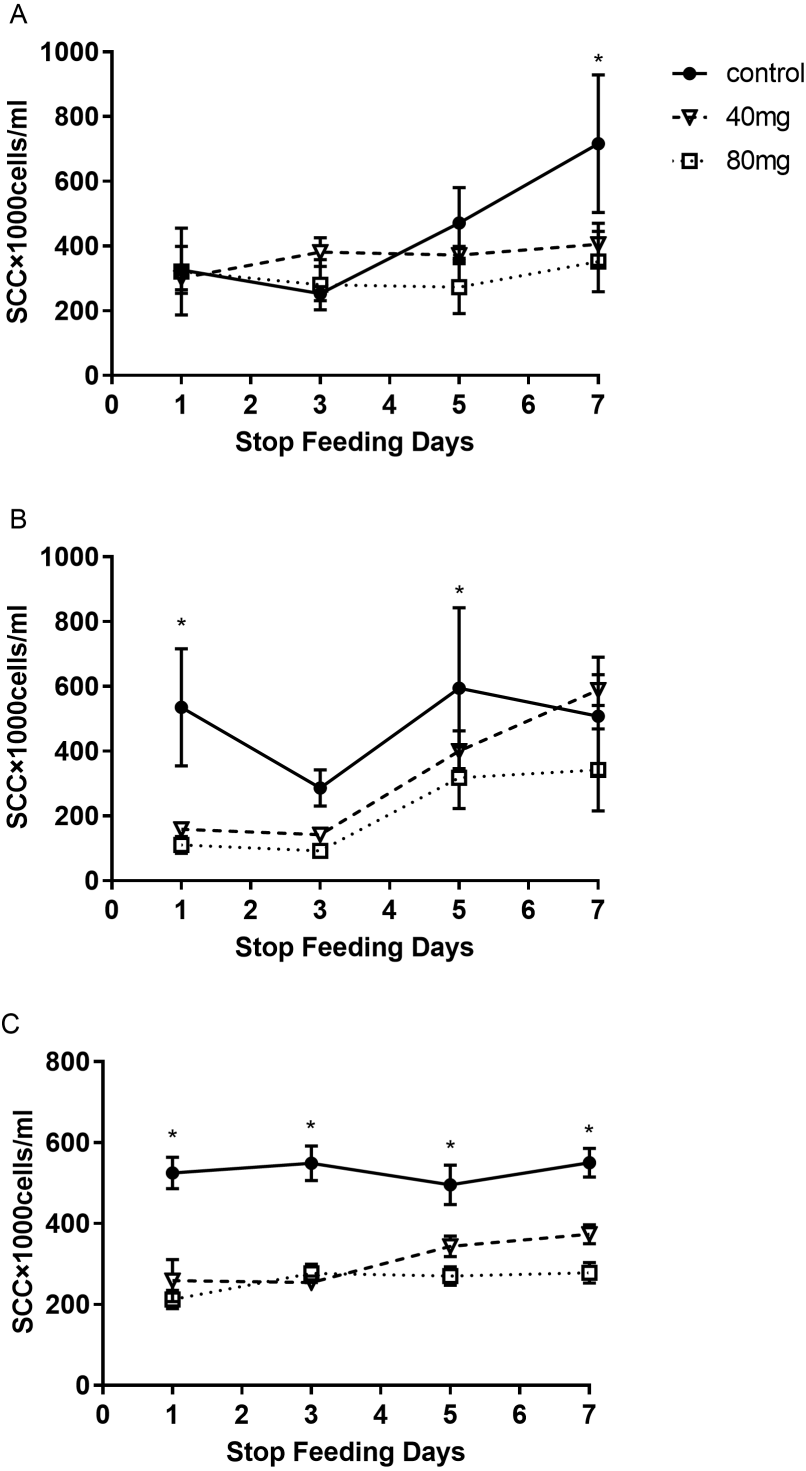
The levels of SCC after termination of RBMF in different groups. (A) Seven-day RBMF group; (B) 14-day RBMF group; (C) 21-day RBMF group. Mean ± SEM (40 mg, *n* = 5; 80 mg, *n* = 5; control, *n* = 5), ^∗^*P* < 0.05.

The results indicated that at day 7 after RBMF feeding, the milk SCC started to decline, but it was not reached the significant difference compared to the controls (Fig. 1A). However, after 14 days of RBMF the milk SCC significantly reduced in both melatonin dose treated groups compared to the control group (*P* < 0.01, Fig. 1B). The similar results were observed after 21 days of RBMF feeding, especially in the 40 mg/cow treated group (*P* < 0.05, Fig. 1C). Interestingly, when the RBMF was terminated, its milk SCC declining effect still lasted for a few days. For example, in 14 days treated groups, the milk SCC declining effect has lasted for 5 days (*P* < 0.05, Fig. 2B) and for the 21 days treated groups it lasts, at least, for 7 days (*P* < 0.05, Fig. 2C).

#### Milk protein

**Figure 3:**
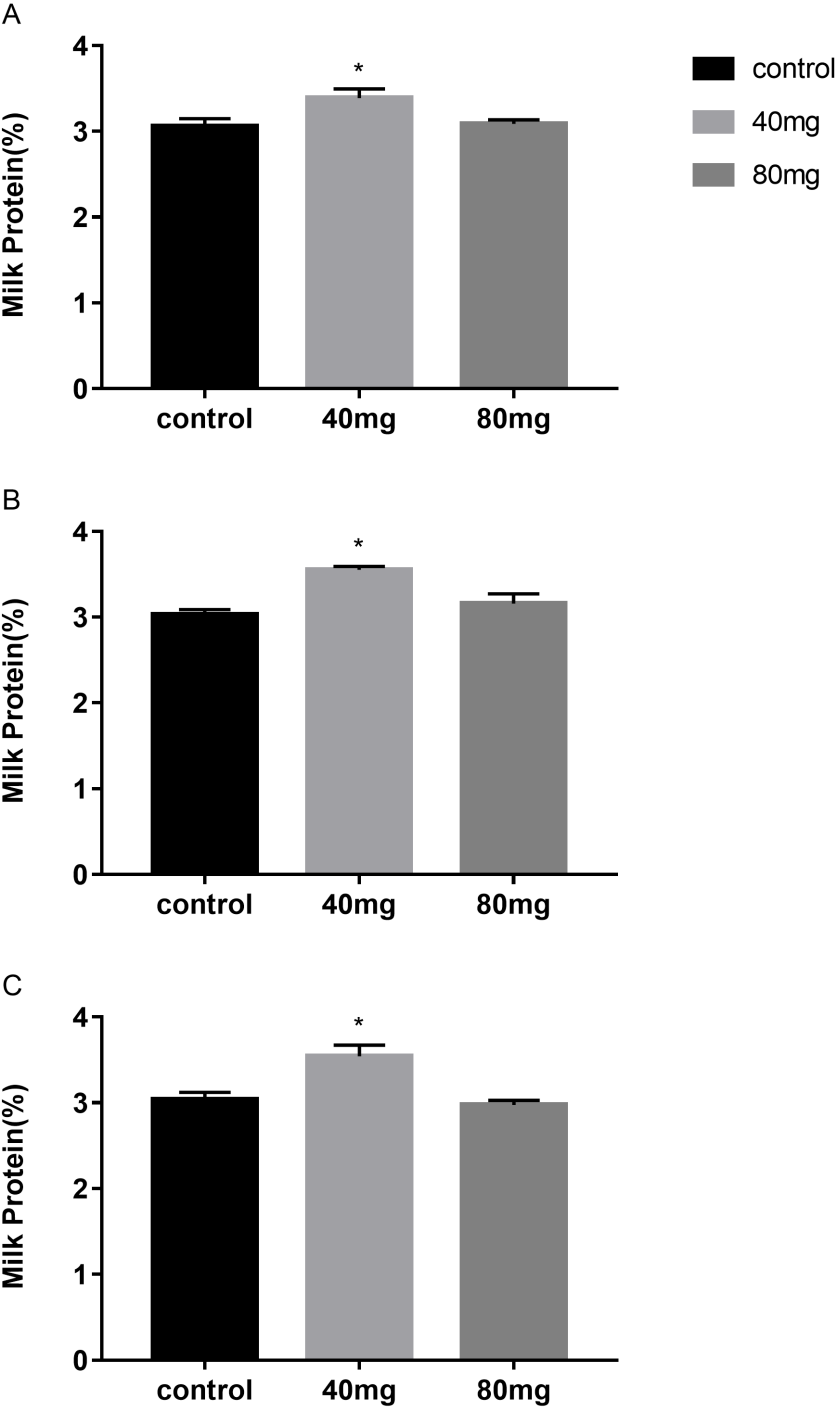
Effects of RBMF on the milk protein in different groups. (A) Milk protein level in RBMF 7-day group; (B) milk protein level in RBMF 14-day group; (C) milk protein level in RBMF 21 -day group. Mean ± SEM (40 mg, *n* = 5; 80 mg, *n* = 5; control, *n* = 5), ^∗^*P* < 0.05.

**Figure 4:**
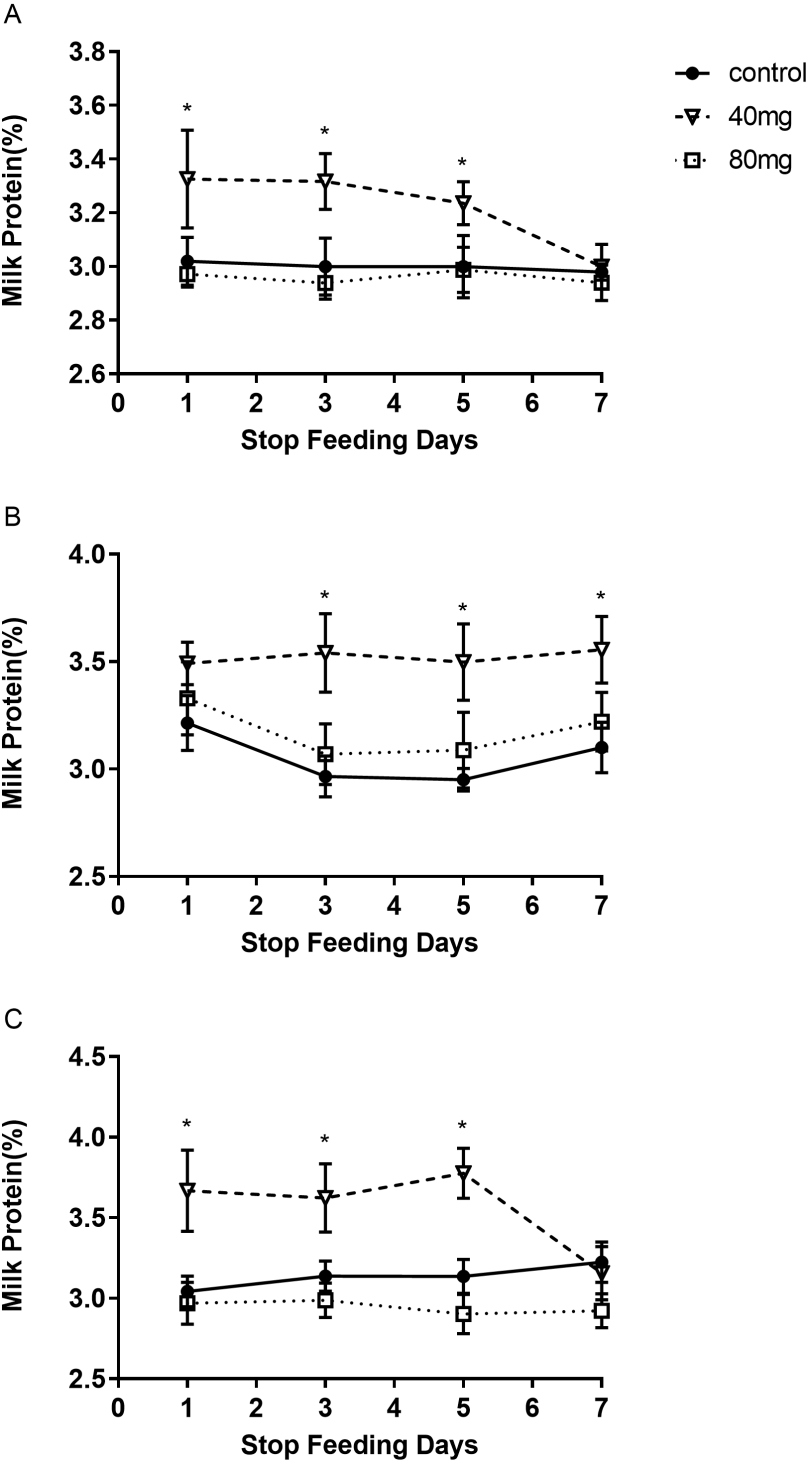
The content of milk protein after RBMF termination in different groups. (A) The milk protein level in the 7-day feeding group; (B) the milk protein level in the 14-day feeding group; (C) the milk protein level in the 21-day feeding group. Mean ± SEM (40 mg, *n* = 5; 80 mg, *n* = 5; control, *n* = 5), ^∗^*P* < 0.05.

The results showed that RBMF at 40 mg group significantly increased the milk protein in 7, 14 and 21 days treated groups, respectively (*P* < 0.05, Fig. 3). The milk protein was 3.39 ± 0.11 in 7-day 40 mg group (Fig. 3A), 3.55 ±  0.05 in 14-day 40 mg group (Fig. 3B) and 3.54 ±  0.04 in 21-day 40 mg group (Fig. 3C), respectively. The milk protein increasing effect was lasted for a few days even after the RBMF was terminated, especially in the 40 mg-treated groups (Fig. 4).

#### Milk fat

**Figure 5:**
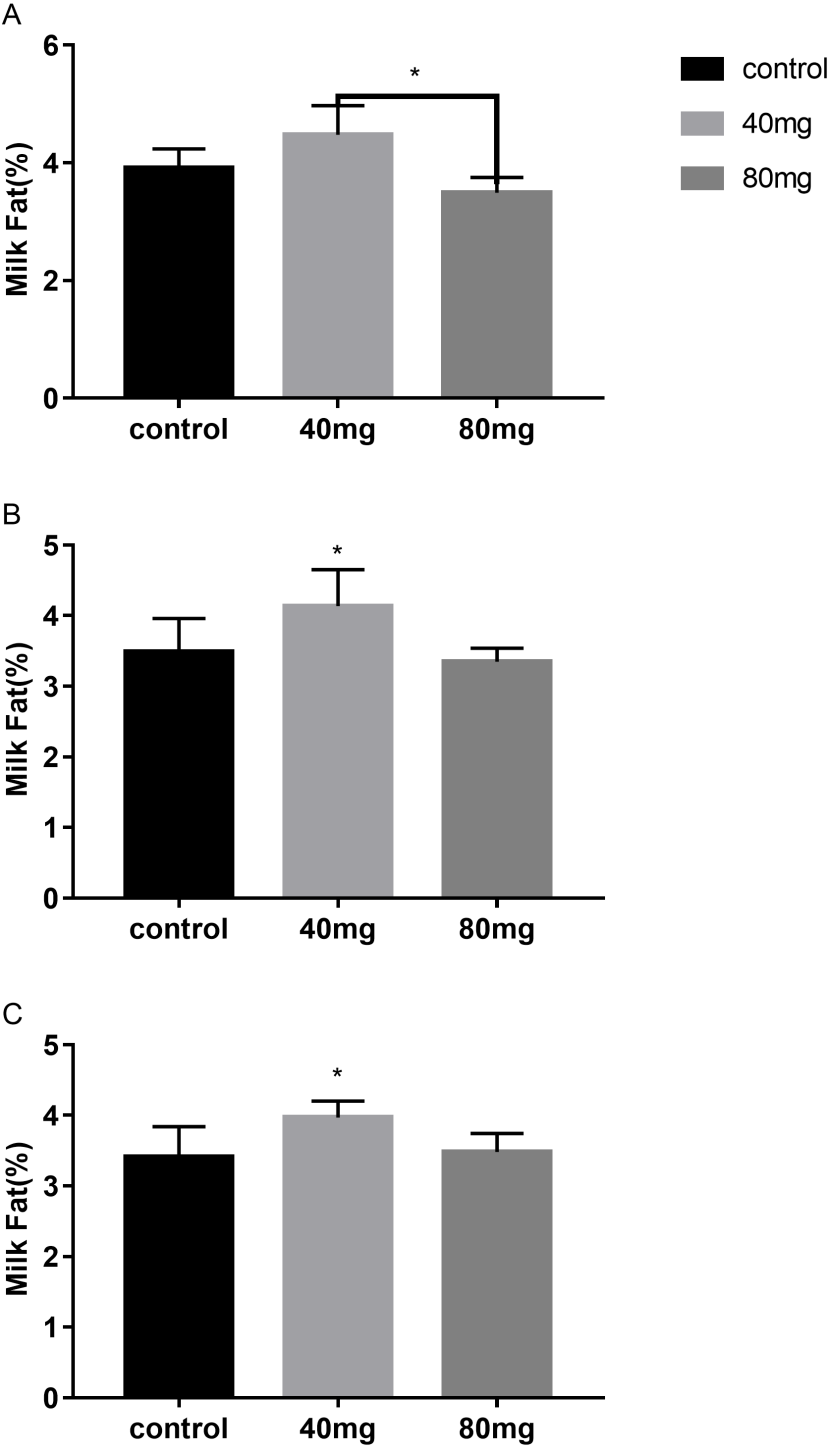
Effects of RBMF on the milk fat content in different groups. (A) Milk fat content in 7-day feeding group; (B) milk fat content in 14-day feeding group; (C) milk fat content in 21-day feeding group. Mean ± SEM (40 mg, *n* = 5; 80 mg, *n* = 5; control, *n* = 5), ^∗^*P* < 0.05.

**Figure 6:**
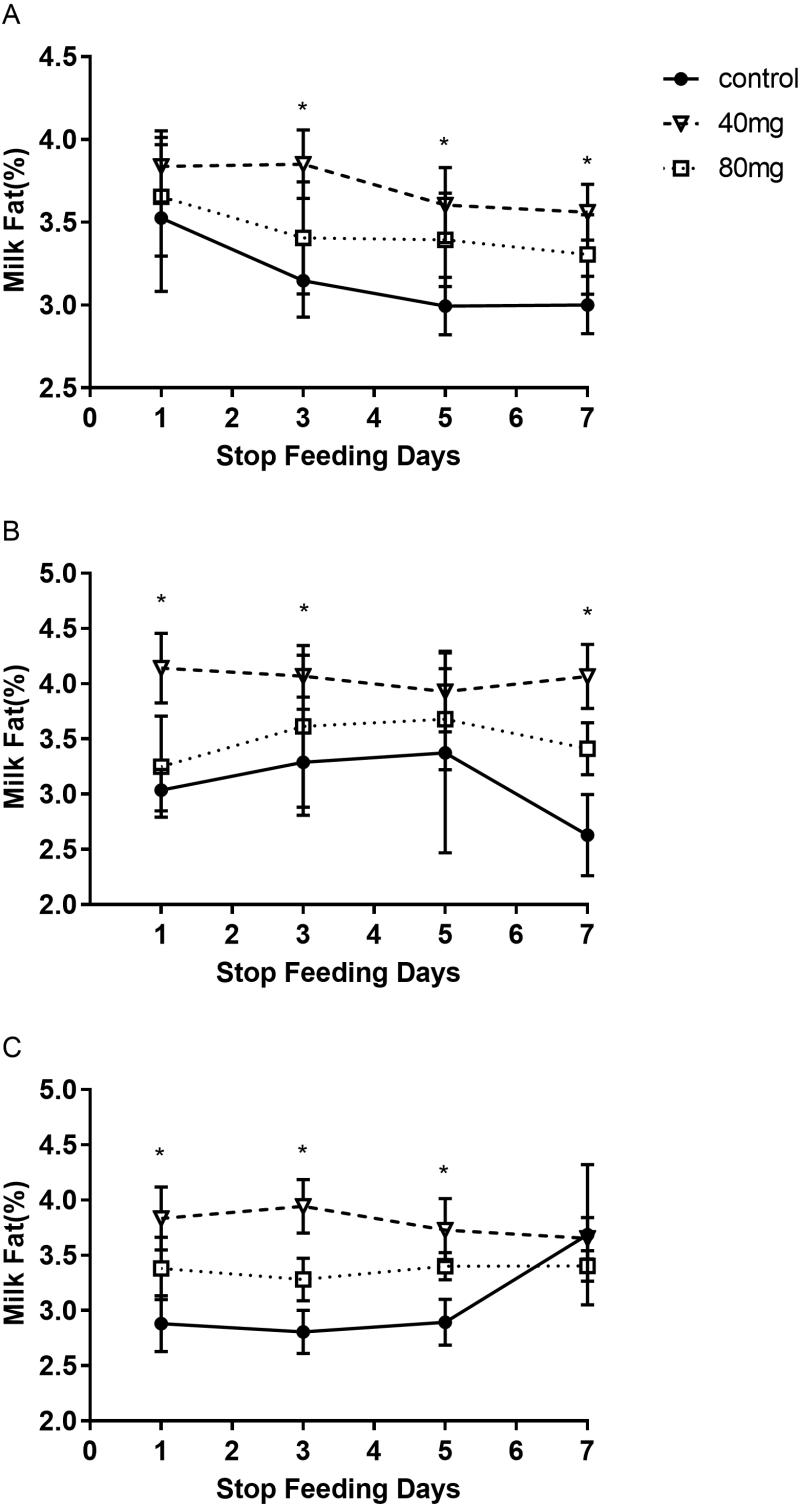
The milk fat content after RBMF termination in different groups. (A) Milk fat content in the 7-day feeding group; (B) milk fat content in the 14-day feeding group; (C) milk fat content in the 21-day feeding group. Mean ± SEM (40 mg, *n* = 5; 80 mg, *n* = 5; control, *n* = 5), ^∗^*P* < 0.05.

As shown in Fig. 5, rumen bypass melatonin feeding in the 40 mg group significantly improved the content of milk fat in 14-day and 21-day groups compared to the control group, respectively (*P* < 0.05). Milk fat contents were 4.13 ± 0.19 in the 14-day 40 mg group (Fig. 5B) and 3.96 ± 0.10 in the 21-day 40 mg group (Fig. 5C), respectively. In addition, this upsurge lasted for several days after the termination of the RBMF in 40 mg group compared to the control group (Fig. 6), especially in 14-day 40 mg group, it was observed for 7 days after the RBMF (Fig. 6B).

**Figure 7:**
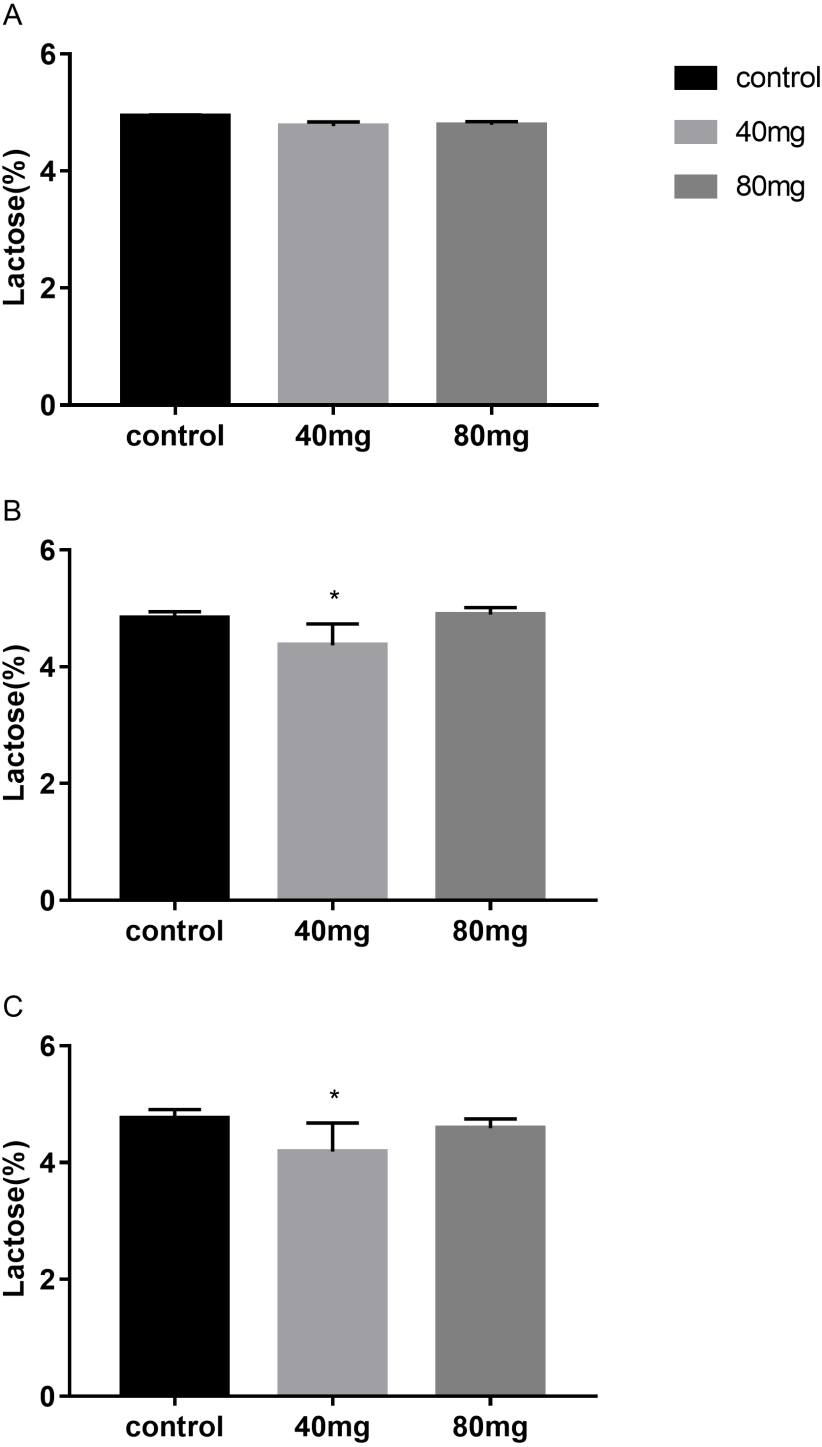
Effects of RBMF on the milk lactose content in different groups. (A) Milk lactose content in 7-day feeding group; (B) milk lactose content in 14-day feeding group; (C) milk lactose content in 21-day feeding group. Mean ± SEM (40 mg, *n* = 5; 80 mg, *n* = 5; control, *n* = 5), ^∗^*P* < 0.05.

#### Lactose

**Figure 8:**
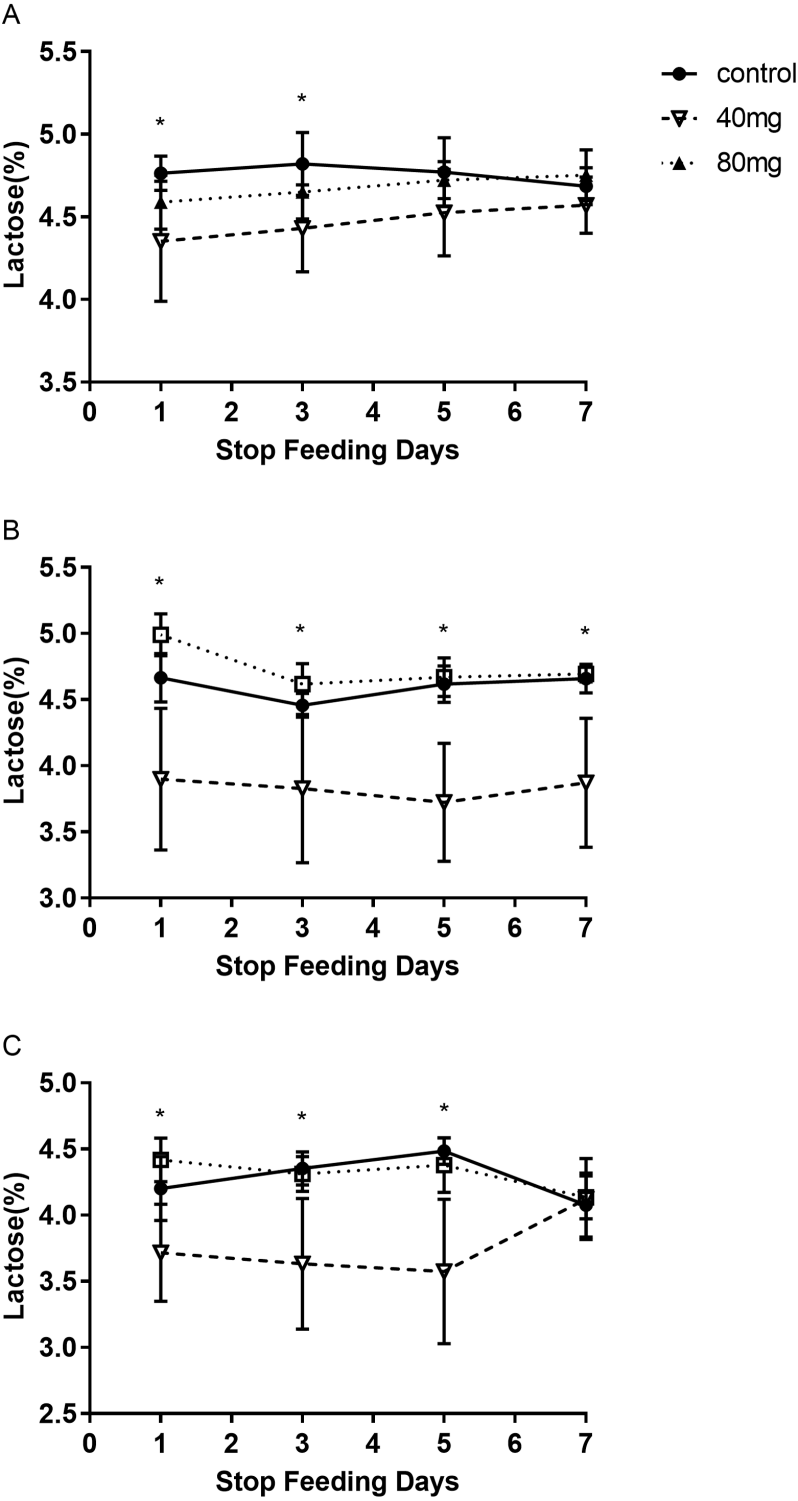
The milk lactose content after RBMF termination in different groups. (A) Milk lactose content in the 7-day feeding group; (B) milk lactose content in the 14-day feeding group; (C) milk lactose content in the 21-day feeding group. Mean ± SEM (40 mg, *n* = 5; 80 mg, *n* = 5; control, *n* = 5), ^∗^*P* < 0.05.

The results showed that RBMF in 40 mg group at 14 days and 21 days significantly reduced lactose content in milk (*P* < 0.05, Fig. 7). The lactose were 4.37 ± 0.14 in 14-day 40 mg group (Fig. 7B) and 4.19 ± 0.10 in 21-day 40 mg group (Fig. 7C). This reduced lactose content lasted for several days after the RBMF termination in 40 mg group compared to the control group (*P* < 0.05, Fig. 8), especially in 14-day 40 mg group, it lasted 7 days after the RBMF termination (Fig. 8B).

#### Milk dry matter

**Figure 9:**
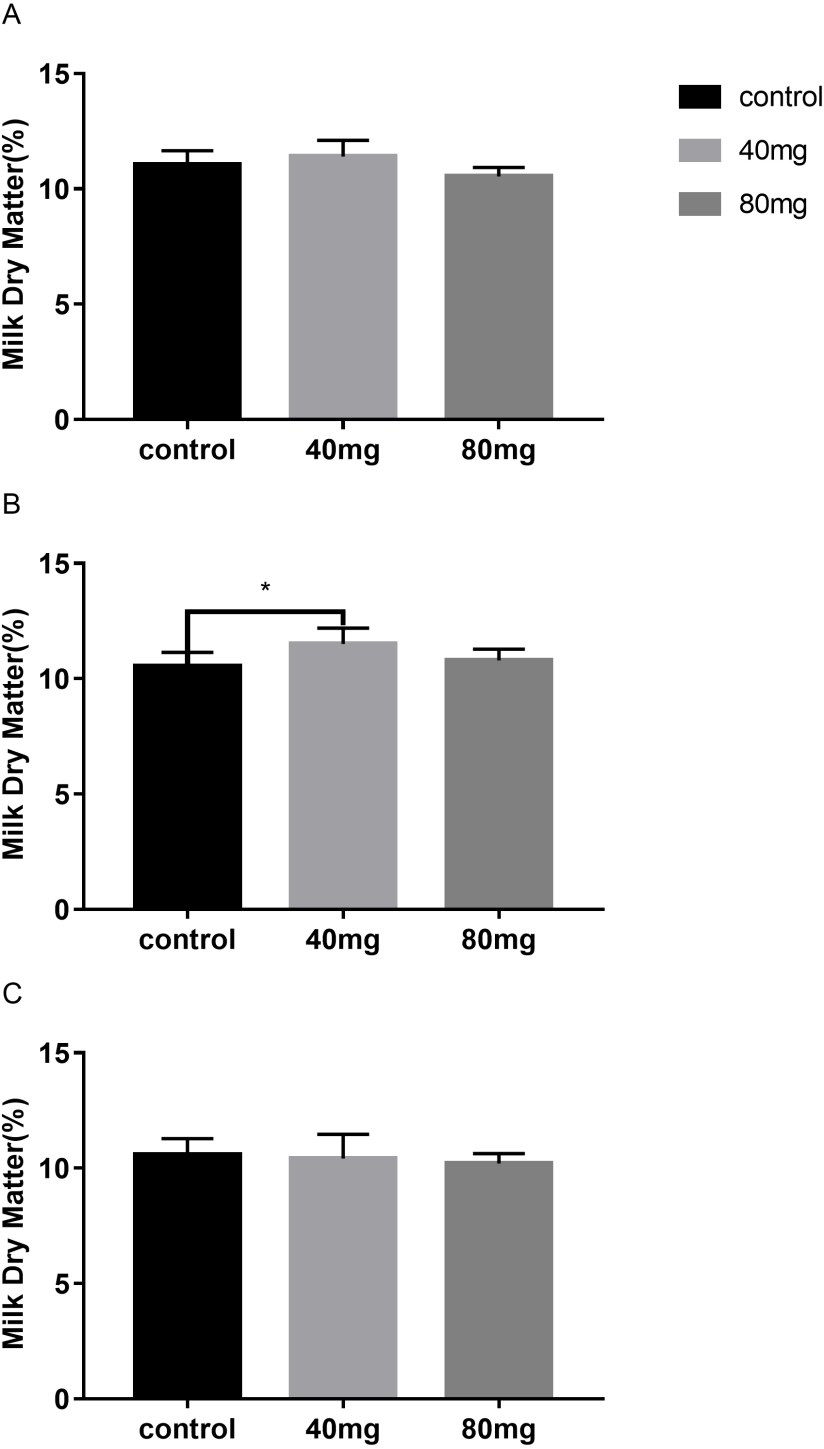
Effects of RBMF on the milk dry matter content in different groups. (A) Milk dry matter in 7-day feeding group; (B) milk dry matter in 14-day feeding group; (C) milk dry matter in 21-day feeding group. Mean ± SEM (40 mg, *n* = 5; 80 mg, *n* = 5; control, *n* = 5), ^∗^*P* < 0.05.

**Figure 10:**
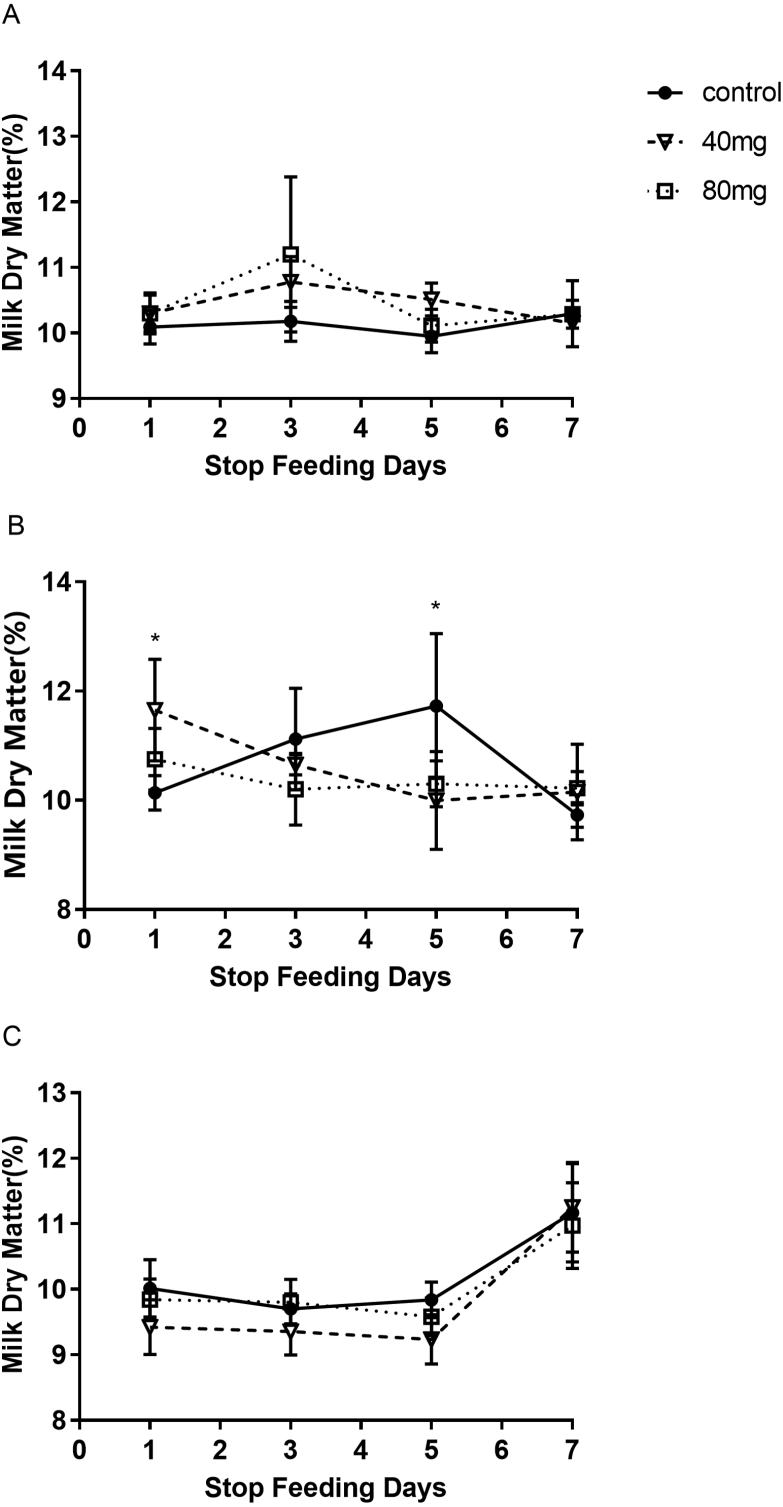
The content of milk dry matter after RBMF termination in different groups. (A) Milk dry matter in the 7-day feeding group; (B) milk dry matter in the 14-day feeding group; (C) milk dry matter in the 21-day feeding group. Mean ± SEM (40 mg, *n* = 5; 80 mg, *n* = 5; control, *n* = 5) ^∗^*P* < 0.05.

As shown in Fig. 9, the content of milk dry matter after 14 days of RBMF feeding in the 40 mg group RBMF was significantly higher than that in the control group (11.50 ± 0.29 vs 10.52  ± 0.19) (*P* < 0.05) (Fig. 9B). However, this increase was not shown a significant difference compared to the control group after the RBMF termination. No significant differences were observed in other feeding groups compared to the control group (Fig. 10).

### Effects of RBMF feeding on melatonin levels in serum and in milk

**Figure 11:**
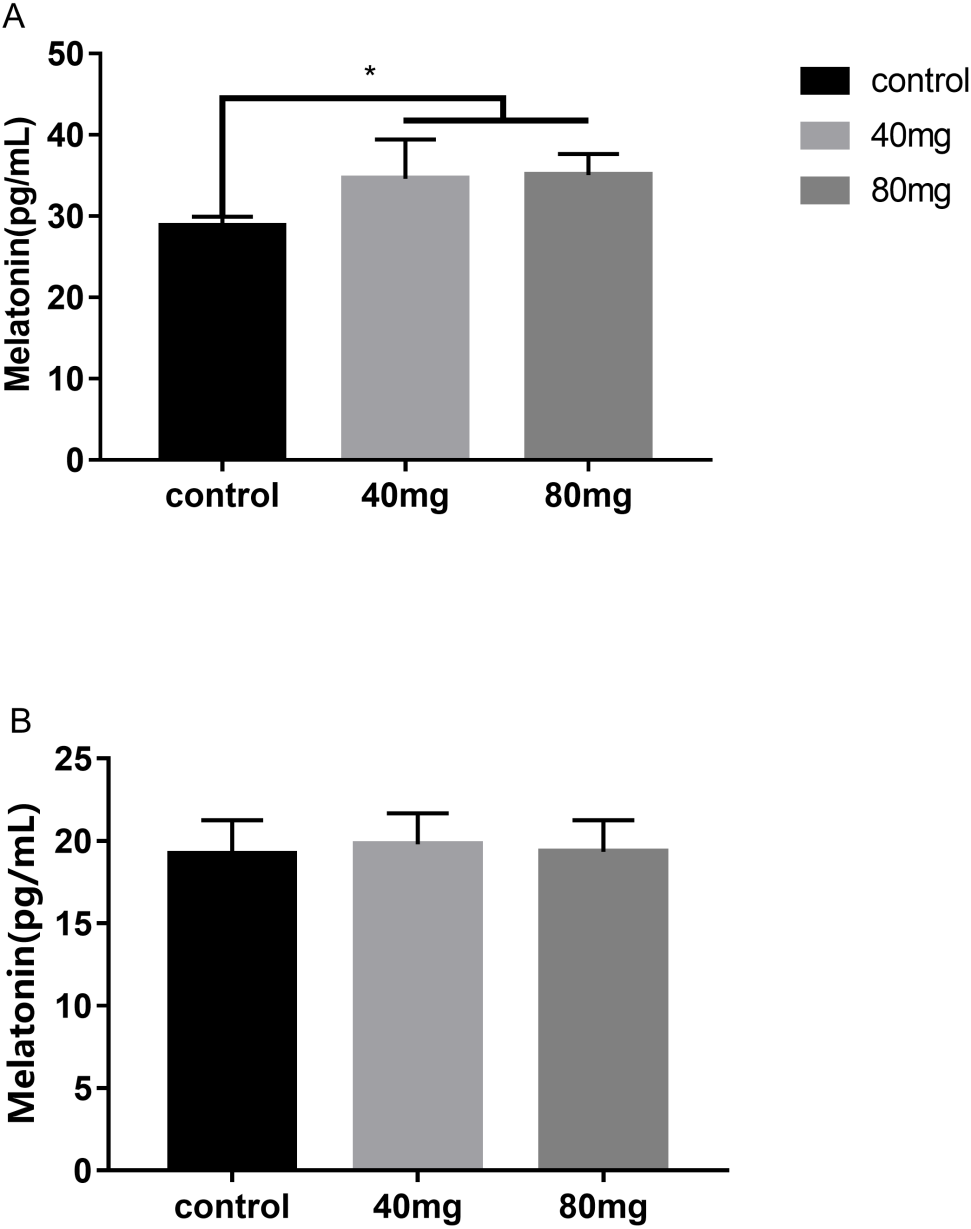
Effects of RBMF on melatonin levels in serum and in milk. (A) Melatonin levels in serum; (B) melatonin levels in milk. Mean  ± SEM (40 mg, *n* = 15; 80 mg, *n* = 15; control, *n* = 5), ^∗^*P* < 0.05.

The results showed that RBMF in both 40 mg/cow and 80 mg/cow groups significantly increased the serum melatonin levels compared to the control group (*P* < 0.05, Fig. 11A) and no significant difference was observed between the two different melatonin doses feeding groups. In addition, RBMF at the current doses was not significantly increase the milk melatonin levels compared to the control group (Fig. 11B).

### Effects of RBMF on serum stress hormone, cortisol level

**Figure 12:**
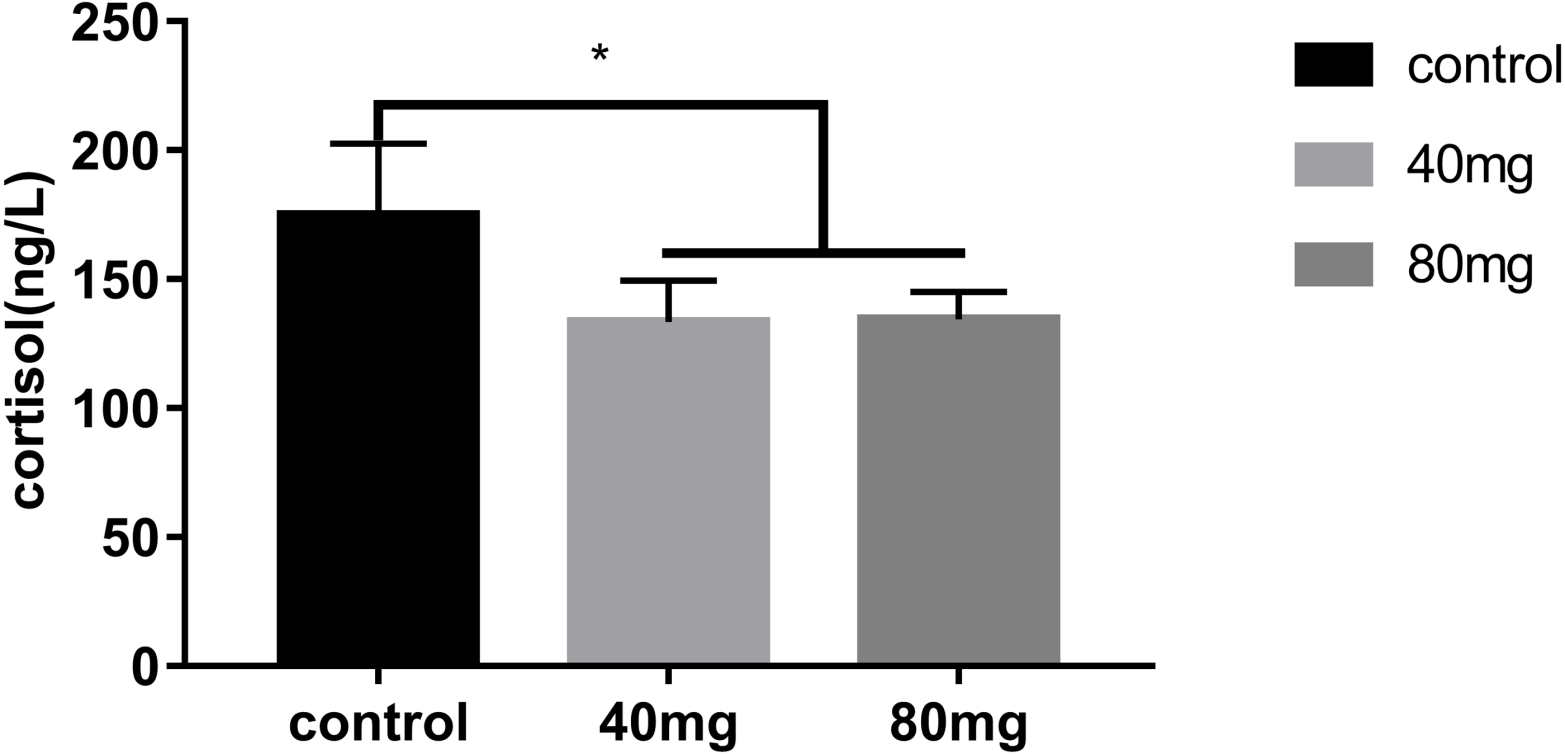
Effects of RBMF on serum cortisol levels. Mean ± SEM (40 mg, *n* = 15; 80 mg, *n* = 15; control, *n* = 5), ^∗^*P* < 0.05.

The results showed that RBMF significantly reduced the serum cortisol level in both 40 mg/cow and 80 mg/cow groups compared to the control group (*P* < 0.05, Fig. 12).

### Effects of RBMF on pro and anti-inflammatory cytokines in the serum and the milk

**Figure 13:**
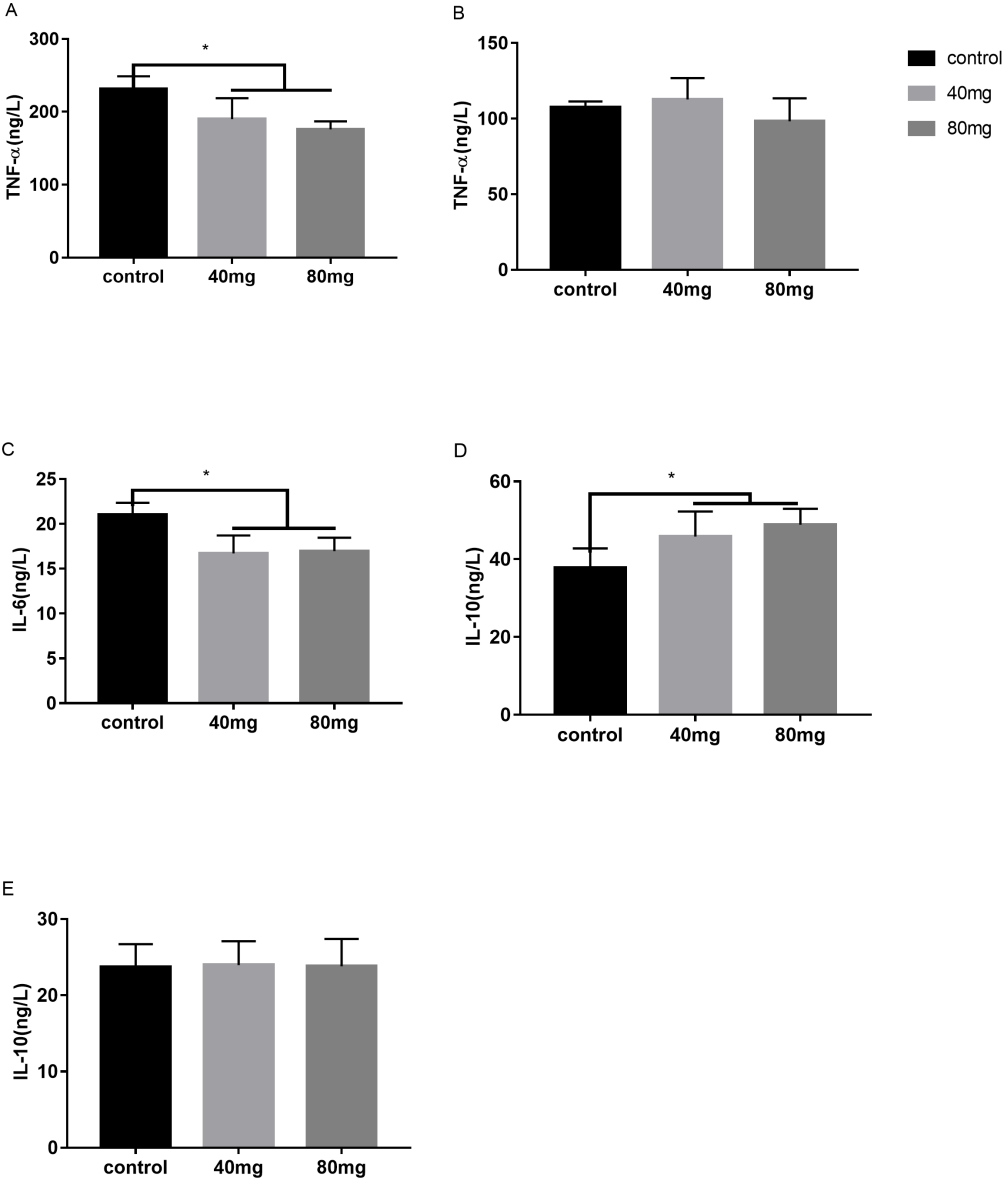
Effects of RBMF on the pro and anti-inflammatory cytokines. (A) TNF-α level in serum; (B) TNF-α level in milk; (C) IL-6 level in serum; (D) IL-10 level in serum; (E) IL-10 level in milk. Mean ± SEM (40 mg, *n* = 15; 80 mg, *n* = 15; control, *n* = 5), ^∗^*P* < 0.05.

These cytokines include tumor necrosis factor alpha (TNF-α), interleukin-6 (IL-6) and interleukin-10 (IL-10). The results indicated that RBMF significantly reduced the serum TNF-α levels in both melatonin doses-feeding groups compared to the control groups (*P* < 0.05, Fig. 13A). However, the milk TNF-α levels was not showed significant differences among all the groups (Fig. 13B). For the proinflammatory cytokine IL-6, RBMF significantly reduced its serum levels in both doses of melatonin feeding groups compared to the control group (*P* < 0.05, Fig. 13C). In contrast, for the anti-proinflammatory cytokine IL-10, RBMF significantly enhanced its serum levels in both doses of melatonin feeding groups compared to the control group (*P* < 0.05, Fig. 13D) while no significant differences of IL-10 levels were observed in the milk among all the groups (*P* > 0.05, Fig. 13E).

## Discussion

Cow mastitis is a frequently encountered problem for dairy farmers. It is manifested by the elevated milk SCC and reduced milk quality (Baro, Perez & Grillo, 2005). Thus, mastitis caused great economic loss for dairy farmers. It has been estimated that the economic cost of mastitis ranges from texteuro61 to texteuro97 per cow with differences among farmers (Hogeveen, Huijps & Lam, 2011). The antibiotics are the common practice to treat mastitis with the obvious effects to reduce the milk SCC (Aungier & Austin, 1987). However, this practice has far-reaching adverse bio-consequences. For example, the residue of antibiotics in the milk can cause serious health problems, especially in children (Chen, Huang & Lin, 2004). The residue of antibiotics excreted from the cows also impact the ecosystem by disturbance of the natural microbiota (Hammer et al., 2016). Thus, the alternative approaches rather than the antibiotics for controlling the cow’s mastitis are not only necessary but urgent.

In our lab, we have first reported that the muscle injection of melatonin significantly reduced the milk SCC in cows with mastitis (Yang et al., 2017). The results are consistent with the observation in goats in which subcutaneous implantation of melatonin also reduced the SCC in their milk (Zinn et al., 1988; Jiménez, Andrés & Sánchez, 2009). As we have realized that the daily melatonin injection is a continuously stressful maneuver to the cows, especially under the high temperature during summer and its prolonged injection also increases the chance of infection for the cows. As to the subcutaneous melatonin implantation, it has its common shortcoming with the difficulty of exact dosing controlling. Thus, the oral application seems a better method for melatonin delivery. Indeed, melatonin is well absorbed by the gut and has high bioavailability after oral application in mammals those are without rumens (Baro, Perez & Grillo, 2005). The cows are ruminants. The challenge of oral application of melatonin in cows is difficult to control its bioavailability because the oral melatonin must pass the rumen first before entering into the body. However, some studies show that rumen can reduce the bioavailability of some nutrients (Blum, Bruckmaier & Jans, 1999; Bach & Stern, 2000; Galbraith et al., 2016). To overcome these obstacles, in the current study, a novel approach was used to deliver melatonin to the cows, that is, melatonin delivery was bypassed the rumen. This approach is referred as “rumen bypass melatonin feeding” (RBMF) (see methods). This approach prevents melatonin to be metabolized by the enzymes and microbes contained in the rumens and promotes its bioavailability in cows. The results showed that RBMF at the dose of either melatonin 40 mg/day/cow or 80 mg/day/cow significantly reduced the milk SCC and improved the milk quality compared to the control group. The potential mechanisms may involve the antioxidant and anti-inflammatory activities of melatonin. Melatonin is a mitochondrial-targeted antioxidant (Tan et al., 2016) and effectively scavenges the ROS generated by mitochondrial metabolism, thus, it suppresses the oxidative stress occurring in the mastitis. Melatonin also up-regulates Nrf2 and heme oxygenase-1 expression in the antioxidant defense pathway and improves dityrosin level and inhibits RNS generation (Yu et al., 2017). The antioxidative stress effect was indicated by the reduced serum stress hormone, cortisol level after RBMF (Fig. 12). The anti-inflammatory activity of melatonin is well documented. Melatonin inhibited LPS-binding protein to CD14–TLR4 in bovine mammary epithelial cells (bMECs) and decreased the expression of LPS-induced proinflammatory factors. Those factors include TNF-α, IL-1 β, IL-6, granulocyte-monocyte colony-stimulating factor, chemokine CC motif ligand2(CCL2), CCL5, serum amyloid A, haptoglobin, C-reactive protein, ceruloplasmin, and *α*-1 antitrypsin and increased anti-inflammatory factors of IL-1Ra and the negative acute-phase proteins(APPs) fibrinogen (Yu et al., 2017). Melatonin reduces the inflammatory process in a variety of ways. It removes the highly toxic hydroxyl radical (-OH), peroxynitrite anion (ONOO-), and hypochlorous acid (HOCl) Reiter et al., 2000), reduces the content of TNF-α, TNF-*γ*, IL-6 and increases the expression of IL-10 and TGF-β (Huang et al., 2019). The anti-inflammatory effects of melatonin are confirmed in the current study. After RBMF, the serum levels of TNF-α and IL-6 were significantly reduced and the level of IL-10, on the other hand, was significantly elevated compared to the control.

In addition to the decreased milk SCC, the nutritional quality of the milk also significantly improved by RBMF. It was reported that melatonin supplementation or different photoperiodic exposure schedules modified feeding habit, milk production and milk composition in cattle and sheep (Eisemann et al., 1984; Sanchez-Barcelo et al., 1991; Auldist et al., 2007; Molik et al., 2011; Hogeveen, Huijps & Lam, 2011; Lacasse, Vinet & Petitclerc, 2014; Yu et al., 2017; Huang et al., 2019). The authors claimed that the increase in milk fat and protein in cows fed with melatonin was unlikely related to changes in nutrient composition of the diet but may relate to the alterations of nutrient digestibility or utilization by the cows (Eisemann et al., 1984). Sanchez-Barcelo et al. (1991) suggested that melatonin feeding could affect progesterone production in cows. The altered progesterone production would modify the rate of intestinal calcium absorption, finally, impacted digestibility and efficiency of nutrient utilization in the cows. In addition, it was reported that short photoperiod improved the feed efficiency of the heifer and increased milk production in the following lactation period (Lacasse, Vinet & Petitclerc, 2014). Molik et al. (2011) found that the subcutaneous implantation of melatonin significantly improved the milk protein content and reduce the lactose and fatty acid content in sheep’s milk. Similar results were reported by Auldist et al. (2007). They found that melatonin also reduced lactose levels and increased fat, protein and casein levels in milk. These observations are consistent with our discovery in the current study, that is, the milk protein, fat and dry matter are increased by 29.8, 18.6 and 9.3%, respectively and milk lactose was reduced by 9.71 percent with RBMF. Importantly, we first found that even after the termination of RBMF, the beneficial effects of this approach on milk quality have lasted, at least, for 3 days to one week. Melatonin has a short half-life around 18∼27 min in lactating cows and goats (Eriksson et al., 1998) and this lasting effect is unlikely attributed to melatonin *per se* but may associate to its indirect effects on gene expressions which are related to the inflammatory responses. Melatonin has the capacity to upregulate gene expressions of anti-inflammatory enzymes and to downregulate the gene expression of pro-inflammatory enzymes (Xia et al., 2012). The exact mechanisms for this lasting effect are warranted to further investigations.

## Conclusions

The new approach of RBMF was as effective as other melatonin delivery methods to reduce the milk SCC and improve the milk quality, and at the same time RBMF dramatically reduced the stressful conditions that occurred in other melatonin delivery methods such as in muscle injection or under skin implantation. Most importantly, the beneficial effects of this approach can last for a quite long period (at least one week) after the treatment, which has not been reported in other methods. The speculated mechanisms are that melatonin protects mammary epithelial cells from inflammation and oxidative stress, and promotes the recovery of mastitis (Yu et al., 2017). Melatonin is an environmentally friendly molecule with no or low toxicity to organisms and the RBMF is invasive and convenient. Based on these factors, we recommend that RBMF can be used in the dairy farmers to replace the antibiotics for treatment of the mastitis. It would decrease the cost for mastitis management and improve the milk quality. RBMF is convenient with remarkable outcomes to improve milk quality and cow’s health.

##  Supplemental Information

10.7717/peerj.9147/supp-1 Supplemental Information 1 DHI and hormone detection raw data

## Additional Information and Declarations

### Data Availability

The following information was supplied regarding data availability: The raw data are available in the Supplementary Files.
